# Influence of Electrical Traps on the Current Density Degradation of Inverted Perovskite Solar Cells

**DOI:** 10.3390/ma12101644

**Published:** 2019-05-20

**Authors:** Hyunho Lee, Changhee Lee, Hyung-Jun Song

**Affiliations:** 1 Department of Electrical and Computer Engineering, Inter-University Semiconductor Research Center, Seoul National University, Seoul 08826, Korea; scopild@snu.ac.kr; 2 Department of Safety Engineering, Seoul National University of Science and Technology, Seoul 01811, Korea

**Keywords:** perovskite solar cells, degradation, impedance, Mott–Schottky plot, temperature analysis

## Abstract

Premature aging of perovskite solar cells (PSC) is one of the biggest challenges for its commercialization. Particularly, PSCs exhibit rapid degradation of photovoltaic parameters under ambient air exposure. To estimate the degradation mechanism of PSC under air exposure, we systematically analyzed the relationship between electrical traps of the PSC and its degradation. After 240 h of air exposure to the PSC, its power conversion efficiency degraded to 80% compared to its initial value. The loss mainly originated from reduced current density, which is affected by traps and carrier transport in the disordered semiconducting layer. Capacitance–voltage plots of the PSC showed that the ionic doping from the perovskite layer caused an increased number of trap sites at the buffer layer. Moreover, the extrapolation of temperature dependent open circuit voltage graphs indicated that the trap sites lead to poor carrier transport by increasing recombination losses in the aged device. Therefore, trap sites arose from the result of ion migration and caused an early degradation of PSC under air exposure.

## 1. Introduction

Perovskite solar cells (PSCs) have been successfully developed based on the superior electrical and optical characteristics of perovskite film. The diffusion length of photo-generated carriers exceeds hundreds of nano-meters in perovskite film [1,2]. In addition, the absorption coefficient of lead iodide base perovskite was reported as 1.5 × 10^4^ cm^−1^ at 550 nm. Thus, the incoming visible light can be absorbed by the thin perovskite layer (less than 1 μm) [3]. As a result of the excellent optical and electrical properties of perovskite film, power conversion efficiency (PCE) has rapidly been improved, reaching 23.2% since the first PSC was reported in 2009 [4,5]. However, perovskite film has an innate weakness to humidity because of its structural ionic bond. Many papers have reported on the stability of PSCs, especially the degradation sources and their effects on the device degradation [6,7,8]. Under exposure to ambient conditions (light, oxygen, moisture), by-products such as methylamine (CH_3_NH_2_), lead iodide (PbI_2_), and iodine (I_2_) are formed as a result of perovskite decomposition [9,10,11,12]. These by-products have undesirable effects (e.g., ionic diffusion from the perovskite layer to other interlayers) on the device performance [13,14,15]. Due to a highly reactive interaction between halide ions and metal electrodes, the ionic by-products, such as I- and Br-, migrate from the perovskite layer to the counter metal electrodes [16,17,18]. The formation of metal-halide compounds is suspected to prohibit charge carrier extraction [19,20]. Furthermore, migration of the organic composition of perovskites, such as methylammonium ion (MA^+^), has been reported as evidence of device degradation [21,22]. Our previous report described the ion-diffusion-induced long term degradation mechanism of PSCs [23]. The study revealed that the burn-in loss of the initial stage (~240 h) mainly originates from the degradation of the perovskite layer. The perovskite film deteriorated by ion diffusion, showing bandgap shrinkage, which was confirmed by electroluminescence data. However, in-depth analysis on the initial burn-in loss of photovoltaic parameters was not clearly elucidated.

Here, we performed electrical analysis on the burn-in loss of the PSCs (CH_3_NH_3_PbI_3_). For 240 h, the photovoltaic performance and the decay trend of each photovoltaic parameter was monitored. One of the main photovoltaic parameters, short circuit current density (*J_SC_*), was identified as the main loss source. Although the absorption of aged film is similar to as-deposited film, the *J_SC_* of the device is significantly reduced. To demonstrate electrical degradation regarding charge transport of the perovskite layer, impedance spectroscopy was introduced. Effects of electrical traps, which originate from the by-products of the decomposed perovskite layer, were investigated. Ionic doping density inside the device, before and after the air exposure test, was estimated by a C-2-V plot (Mott–Schottky), which explains the ion diffusion related device degradation. Moreover, the built-in voltage of PSCs regarding device degradation time was monitored to estimate carrier transport kinetics. Furthermore, the temperature dependence of the open circuit voltage (*V_OC_*) of PSCs was evaluated to derive the unique charge recombination kinetics with respect to device degradation. Employing these electrical analyses, the origin of early degradation of PSC under air exposure was systematically investigated.

## 2. Materials and Methods 

PbI_2_ (99.9985%) was purchased from Alfa-Aesar (Ward Hill, MA, USA). Methylammonium iodide (MAI, 99.5%) was purchased from Xian Polymer Light Technology (Shaanxi, China). [6,6]-Phenyl-C71-butyrate (PCBM) was purchased from 1-Material (Quebec, Canada). Dimethylformamide (DMF), dimethyl sulfoxide (DMSO), isopropyl alcohol (IPA), and chlorobenzene (CB) were purchased from Sigma-Aldrich (Saint Louis, MI, USA). All materials were used as received without any purification. 

Indium tin oxide (ITO) coated glasses were cleaned with acetone, IPA and de-ionized (DI) water. The cleaned substrates were exposed to UV-Ozone for 15 min. Poly(3,4-ethylenedioxythiophene) polystyrene sulfonate (PEDOT:PSS, AI4083) was spin-coated (3500 rpm, 30 s) and annealed (130 °C, 25 min). Substrates were transferred to a glovebox. Perovskite films were fabricated by the sequential deposition method. PbI_2_ solution (1.2M in mixture of DMF and DMSO (9:1)) was spin-coated (3000 rpm, 30 s). MAI solution (50 mg/ml in IPA) was spin-coated (3000 rpm, 30 s) and annealed (100 °C, 120 min.). The thickness of the perovskite layer was about 400 nm. PCBM solution (25 mg/ml in CB) was spin-coated (2000 rpm, 40 s). PCBM coated substrates were kept in a dry box (relative humidity less than 10%) overnight. Substrates were transferred to a vacuum chamber and the Ag electrode was thermally evaporated under 10^−6^ Torr.

For device characterization, a 300W Xenon lamp based solar simulator (Newport 91160A, Newport, CA, USA) and a Keithley 237 (Cleveland, OH, USA) source measurement unit under AM 1.5G 1-sun illumination were used. Light intensity was controlled by optical density filters. Impedance spectroscopy was measured by WAYNE KERR 6500B (Bognor Regis, UK) under dark conditions. The frequency was set to 1 kHz. Temperature was controlled by a LakeShore 331 temperature controller (Carson, CA, USA) and a Suzuki Shokan helium compressor unit (C100G, Tokyo, Japan). The device active area, which is the cross-sectional area of ITO and the Ag electrode, was 0.02 cm^2^. The UV-vis absorbance was measured by HITACHI U-2900 (Hong Kong, China). 

## 3. Results and Discussion

**Figure 1:**
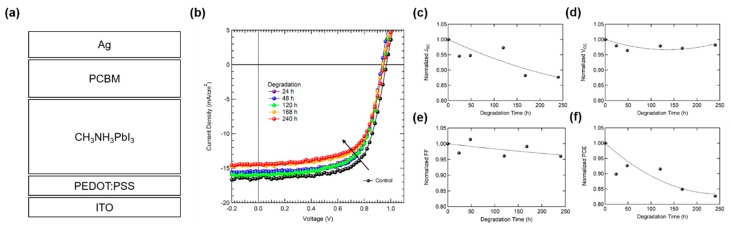
(**a**) Device structure of PSCs (**b**) *J*–*V* curve for each device degradation points under 1 sun condition. Devices were stored in ambient air without any light exposure during device degradation time. Normalized decay trends of each photovoltaic parameter (**c**) *J_SC_*, (**d**) *V_OC_*, (**e**) FF and (**f**) PCE.

**Figure 2:**
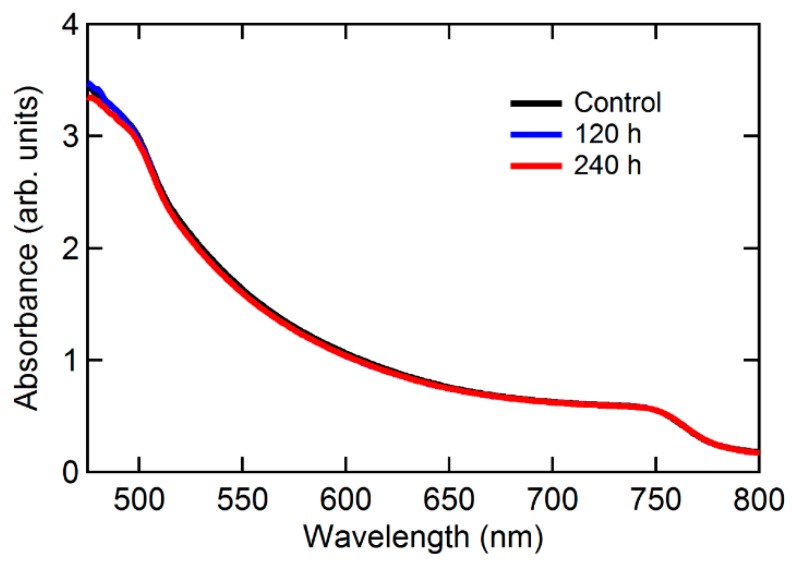
The UV-vis absorbance spectrum for control, 120 h and 240 h degraded perovskite films. Perovskite films were deposited on the PEDOT:PSS coated substrates.

**Figure 3:**
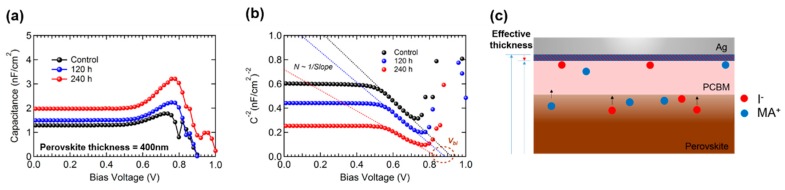
(**a**) *C–V* plot and (**b**) Mott–Schottky plot for the control, 120 and 240 h air exposed PSCs. (**c**) Schematic for the effective thickness decrease by device degradation.

Figure 1a shows the device structure of PSCs. Figure 1b shows current density (*J)*–voltage (*V)* plots of PSCs with respect to air exposure time. Photovoltaic parameters from each device are summarized in Table 1. The decay trends of each parameter are shown in Figure 1c–f. After 240 h of air exposure, the degradation of PCE mainly originates from the degraded *J_SC_*, while the change of *V_OC_* and FF (Fill factor) was not distinctively observed. Normally, two factors affect *J_SC_* in the PSCs: (i) absorption loss from a damaged active layer, and (ii) change of carrier transport dynamics (e.g., traps, band gap, mobility and so on). The absorbance of perovskite film before and after the air exposure test was measured to investigate morphological or optical degradation of perovskite film. Figure 2 is the absorbance of fresh and air exposed perovskite films (120 and 240 h), respectively. The absorbance is in the same range independent of air exposure time, despite significantly reduced *J_SC_* after the test. The reduction in *J_SC_* is irrelevant to the degradation of absorbance. Thus, the formation of electrical traps by device degradation should be investigated. To investigate the variation of electrical characteristics (carrier transport, charge recombination) by device degradation, impedance spectroscopy was measured (Figure 3). Figure 3a shows a *C*–*V* plot of the device with respect to the air exposure time. The capacitance value increased from the control device to the 240 h air exposed device. Capacitance is described as Equation (1)

(1) $$C=\frac{\varepsilon_{0}\varepsilon_{r}A}{d}$$

where ε_0_ is the dielectric constant of the vacuum, ε_r_ is the relative dielectric constant of the doped semiconductor, A is the area and d is the effective thickness between two electrodes. The capacitance is inversely proportional to the thickness when ε_0_, ε_r_ and A are unchanged. Previous reports about the degradation of PSCs showed that ion diffusion from the perovskite layer to other interface layers is the main degradation mechanism of PSCs [15,23]. As the device is degraded, I^−^ and MA^+^ ions migrate and accumulate under the metal electrode. Hence, the effective thickness between each electrode (ITO and Ag) of the device can be thinner as the device is degraded. A schematic for ion diffusion related effective thickness of the device is shown in Figure 3c. According to Equation (1), reduction of the thickness results in an increase in capacitance. Thus, the increase in capacitance correlated with air exposure time is the direct evidence of ion diffusion from the perovskite layer as a result of device degradation (please see Figure 3a). Figure 3b shows a *C^−^*^2^–*V* plot which is the so-called Mott–Schottky plot. PSCs operate under the *p-n* junction typed structure [24]. The doping density inside of the device has considerable impact on the device performance. Here, the doping density means the amount of impurities created by external degradation factors. From the results of the *C*–*V* measurement, the created by-products (diffused iodide and MA ions) owing to device degradation can be applied as a dopant. The doping density N follows the Equation (2)

(2) $$N=\frac{2}{q\varepsilon A}{\left(\frac{\mathrm{dC}{\left(V\right)}^{-2}}{\mathrm{dV}}\right)}^{-1}$$

where q is the elemental charge and ε is the dielectric constant.

From Equation (2), the slope of the Mott–Schottky plot is inversely proportional to N. We calculated the value of each slope from Figure 3b. The inverse slope (1/Slope) of 240 h of air exposed PSC (1.2 × 10^−18^) is one order higher than those of 120 h air exposed (7.9 × 10^−19^) and as-fabricated PSCs (7.0 × 10^−19^). The doping density of PSC increased as the device is stored at an ambient condition. Hence, *C–V* analysis confirms ion diffusion from the perovskite layer as a result of device degradation. 

**Figure 4:**
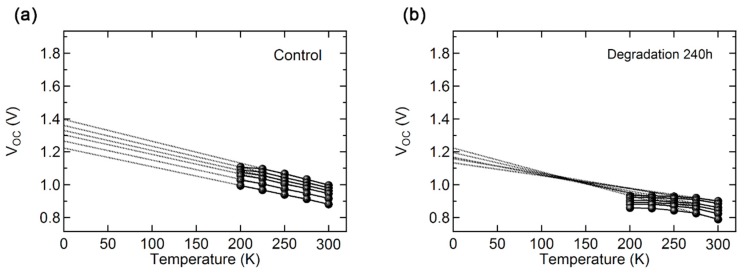
Temperature dependent *V_OC_* plot with different light intensity for (**a**) control device and (**b**) 240 h degraded device. Light intensity varies from 2 to 100 mW/cm^2^. *V_OC_* increases by increasing light intensity. The dashed line represents extrapolation of the temperature dependent *V_OC_* data. The data points for the extrapolation range from 250 K to 300 K.

Moreover, the extrapolation from the *C^−^*^2^–*V* plot indicates built in voltage (*V_bi_*) of PSC, which is related to the depletion region length. In the device with high *V_bi_*, its depletion region, where photo-generated carriers preferentially move to their counter electrodes, is larger than that of the device with low *V_bi_*. Normally, a device with a large depletion region has the advantages of being able to separate excitons, transport charges, and collect photo-generated carriers [25,26]. The obtained *V_bi_* of the as-fabricated device (0.92 *V*) is higher than those of air exposed devices (120 h: 0.88 *V* and 240 h: 0.84 *V*). The result implies that the charge transport property is reduced as a result of device degradation in the air exposed device. The temperature dependent *V_OC_* of PSC clearly reveals that the deteriorated electrical properties of the degraded device lead to reduction of its *J_SC_*. Figure 4 shows the temperature-dependent *V_OC_* plots for different light intensities. Light intensity dependent VOC has been reported to study charge recombination processes [27,28]. Under open-circuit conditions, Equation (3) applies:

(3) $$\frac{{\mathrm{dV}}_{\mathrm{OC}}}{\mathrm{dT}}=\frac{\left(\frac{E_{g}}{q}-V_{\mathrm{OC}}+\frac{\mathrm{kT}}{q}\alpha \right)}{T}$$

Here E_g_ is the band gap of the perovskite film, kT/q is the thermal voltage, and α describes the second-order additional temperature dependence, which includes the np junction product [29]. At T = 0 K, from Equation (3):

(4) $$V_{\mathrm{OC}}=\frac{E_{g}}{q}+\frac{{\mathrm{kT}}_{\mathrm{av}}}{q}\alpha$$

where T_av_ is the average temperature over the given range. The first term of Equation (4), $\frac{E_{g}}{q}$, is related to the absorber recombination mechanism because it only contains the term of bandgap of the perovskite film, and the second term, $\frac{{\mathrm{kT}}_{\mathrm{av}}}{q}\alpha$, is related to other recombination processes, such as those occurring at the *np* junction because it contains a second-order additional temperature dependence term. The first term and the second term in Equation (4) could be the indicator for assigning the dominating type of recombination. If the recombination process inside the perovskite was dominated by the defect states inside the perovskite layer, extrapolation of the *V_OC_* for different light intensities converges to the same point at T = 0 K because other recombination process, which affect *np* junction related recombination, could be neglected. However, if other recombination mechanisms are dominant, the second term varies with different light intensities, and the extrapolations of the *V_OC_* do not converge at T = 0 K. The dashed lines of Figure 4 show the extrapolations of the light intensity and temperature-dependent *V_OC_*. The control device has no defects inside the perovskite layer or the PCBM-related interfacial layer. Because the density of the trap states of the perovskite film is low, other recombination mechanisms were relatively dominant even though the total trap-induced recombination was very low. Thus, the *V_OC_* extrapolation for the control device did not converge to a single point (Figure 4a). On the other hand, after 240 h of air exposure, the convergence of *V_OC_* extrapolation was distinctively observed (Figure 4b). This means that the degradation of the perovskite film generated the defect states inside the perovskite layer, acting as a recombination center. 

## 4. Conclusions

For the initial stage (~240 h) of PSCs degradation, ion-diffusion-induced electrical trap density decides the device performance. Optically unchanged perovskite film regarding degradation time explains that the device degradation originates from the electrical degradation of the perovskite film. The ion diffusion from the perovskite film results in the accumulation of by-products under the Ag electrode, which decreases the effective thickness of the device. Hence, the capacitance of the device increased by the device degradation. The diffused ions act as a dopant, which increases electrical trap density and reduces the *V_bi_*. As a result, carrier transport was inhibited and the formation of an electrical trap can be speculated to be the main loss mechanism in the initial degradation stage.

## Figures and Tables

**Table 1:** Photovoltaic parameters of PSCs regarding degradation time.

Sample	Control	24 h	48 h	120 h	168 h	240 h
*J_SC_* (mA/cm^2^)	16.4	15.6	15.6	16.0	14.5	14.4
*V_OC_* (V)	0.97	0.95	0.93	0.95	0.94	0.95
FF (%)	66.7	64.8	67.6	64.1	66.1	64.0
PCE (%)	10.6	9.5	9.8	9.7	9.0	8.7
